# Thyrotrophin receptors, tumour radioiodine concentration and thyroglobulin secretion in differentiated thyroid cancers.

**DOI:** 10.1038/bjc.1985.225

**Published:** 1985-10

**Authors:** C. J. Edmonds, J. C. Kermode

## Abstract

Tumour radioiodine concentration has been compared with serum thyroglobulin (Tg) and, in a few cases, with tumour complement of thyrotrophin receptors in patients with differentiated thyroid carcinoma. All tumours examined possessed TSH receptors. In most the complement was similar to that of normal thyroid tissue although all but one of the tumours had no detectable 131I concentration in vivo even with excess TSH stimulation. Elevated serum Tg (patient taking T4 in suppressive dose) was generally associated with tumours which had 131I concentrating function when stimulated by excess TSH. Some patients, however, had high serum Tg concentration but only low or indetectable tumour 131I uptake. We conclude that (a) measurement of tumour TSH receptor complement is unlikely to be useful in clinical management as tumours which do not significantly concentrate 131I in vivo may have a normal TSH receptor complement and (b) the capacity to secrete Tg is usually associated with 131I concentration but quantitatively the relationship varies considerably between tumours.


*Br. J. Cancer* (1985), **52**, 537–541

### Thyrotrophin receptors, tumour radioiodine concentration and thyroglobulin secretion in differentiated thyroid cancers

C.J. Edmonds[1] & J.C. Kermode[2]

[1]*Endocrinology Research Group, Clinical Research Centre, Watford Road, Harrow, Middlesex and*
[2]*Department of Radiotherapy and Oncology, University College Hospital, London, WC1E 6AU, UK.*

**Summary** Tumour radioiodine concentration has been compared with serum thyroglobulin (Tg) and, in a few cases, with tumour complement of thyrotrophin receptors in patients with differentiated thyroid carcinoma.

All tumours examined possessed TSH receptors. In most the complement was similar to that of normal thyroid tissue although all but one of the tumours had no detectable $^{131}$I concentration *in vivo* even with excess TSH stimulation.

Elevated serum Tg (patient taking T4 in suppressive dose) was generally associated with tumours which had $^{131}$I concentrating function when stimulated by excess TSH. Some patients, however, had high serum Tg concentration but only low or indetectable tumour $^{131}$I uptake.

We conclude that (a) measurement of tumour TSH receptor complement is unlikely to be useful in clinical management as tumours which do not significantly concentrate $^{131}$I *in vivo* may have a normal TSH receptor complement and (b) the capacity to secrete Tg is usually associated with $^{131}$I concentration but quantitatively the relationship varies considerably between tumours.

For the effective use of $^{131}$I in the treatment of thyroid cancer, adequate organification of the radioisotope by tumour tissue is essential. Most thyroid tumours even though differentiated, are usually nonfunctional initially when normal thyroid tissue is still present. Subsequently a tumour often develops the ability to concentrate $^{131}$I although usually only if stimulated by supranormal levels of TSH (Edmonds *et al.*, 1977). Unfortunately there is no satisfactory way of predicting at an early stage whether a particular tumour will eventually develop sufficient capacity for $^{131}$I concentration. Thyroglobulin production and the presence of TSH receptors on the plasma membranes are two properties indicative of potentially functioning tissue and we have compared these characteristics with the radioiodine uptakes observed during treatment.

### Materials and methods

All patients attended the Thyroid Clinic in the Department of Radiotherapy and Oncology at University College Hospital and were subsequently on long term follow up. The treatment protocol was initial thyroidectomy with removal of as much tumour as possible followed by therapy and test doses of $^{131}$I as previously described (Edmonds *et al.*, 1977). Patients received T4 daily in amount usually 200–300 μg sufficient to suppress the TSH response to TRH. The T4 was stopped 4 weeks before an $^{131}$I dose. Treatment and test doses of 5.5 GBq (150 mCi) and 185 MBq (5 mCi) of $^{131}$I respectively were used. A whole body profile scanner based on the design of Corbett *et al.* (1956) but having a sodium iodide scintillator with a rectangular slit collimator perpendicular to the long axis of the patient was used to estimate the amount of $^{131}$I concentrated at any site. Scanning by gamma camera was also carried out over appropriate regions. Measurements were done at 2 and at 5 to 7 days after the $^{131}$I administration; the delayed measurements allowed excretion of $^{131}$I-iodide so that low levels of functional activity could be determined. The lower limit of sensitivity of localized concentrations detectable was 0.2–0.8 MBq (5–20 μCi).

Fresh human thyroid tissue, tumour and/or lymph nodes were obtained at operation and subsequently classified into their pathological type on the basis of histological examination (kindly performed in the Department of Pathology, University College, London, School of Medicine). Specimens were, in addition, placed on ice and membrane extracts prepared which were later used for measurement of TSH binding capacity and for assessment of binding characteristics: the methods were as those previously described in detail (Kermode & Thompson, 1980; Kermode *et al.*, 1981). Normal thyroid tissue (normal on the basis of histology) was obtained where feasible by

Correspondence: C.J. Edmonds.
Received 11 March 1985; and in revised form 12 June 1985.

© The Macmillan Press Ltd., 1985

dissecting it from abnormal regions in the specimens removed from these and other patients. Most of our studies have been with bovine TSH (bTSH) but a small amount of human TSH has also been available to allow us to compare average binding percentages. Serum thyroglobulin (Tg), measured using a double antibody radioimmunoassay, was determined periodically, usually every 6 months or yearly in all patients attending the Clinic. Antiserum to human Tg was raised in rabbits and used at a final dilution of 1 in $10^5$. Donkey anti-rabbit serum was used as the second antibody. Fifty $\mu$l of $^{125}$I-Tg was added to tubes containing 100 $\mu$l of serum or standard and 110 $\mu$l of antiserum. Twenty-four hours after adding the label, tubes were centrifuged, the supernatant removed and precipitates counted. The Tg assay was sensitive to a limit of about 5 $\mu$g l$^{-1}$. In individuals without thyroid disease, Tg infrequently exceeded 50 $\mu$g l$^{-1}$.

The results are given as mean with standard deviation. The expression of binding capacity in terms of g.equiv (gram equivalent of membranes) relates to an amount of membranes which would be obtained from 1 g (wet weight) of chopped tissue (Smith & Hall, 1974).

### Results

### *TSH binding*

The tumours of all the six patients with differentiated cancer had demonstrable specific binding of $^{125}$I-labelled bTSH and, on most, the binding percentage differed little from the normal value (Table I). The lowest binding was in a tumour of patient 5 but this tumour was also less well differentiated than the others. Of the six patients studied, one (1a, b, Table I and Figure 1) provided two specimens, the first obtained at the initial thyroidectomy, the second two and a half years later when recurrent lymph nodes were removed. The two specimens had similar histological appearance and bTSH binding.

The binding of human TSH (hTSH) was also examined in all of the samples. The general pattern of variation was similar but the binding percentage was considerably less. Thus for the normal tissues average binding was 33.6±9.9% using bTSH compared with 7.9±3.2% using hTSH; for the differentiated tumours, binding averaged 30±7.1% for bTSH compared with 8.2±2.1% for hTSH. Similar differences in binding have been previously

**Table I** Binding characteristics of the TSH receptor, tumour $^{131}$I concentration and serum thyroglobulin (Tg) concentrations in patients with differentiated thyroid cancer

| | | | | *Binding characteristics* | | | | |
|---|---|---|---|---|---|---|---|---|
| Patient | Sex & age (*y*) | *Tumour pathology*[a] (*TNM stage*) | *b TSH binding* (*% age*) | *Capacity* (*pmol q$^{-1}$ equiv*) (*membranes*) | *Affinity* (*l mol$^{-1}$ × 10$^{-9}$*) | *$^{131}$I concentration* (*% dose g$^{-1}$*) | *Serum Tg* *on T4* (*µg l$^{-1}$*) | *off T4* |
| 1a | F 20 | Papillary (p) | 38.1 | 2.08 | 6.9 | | | |
| b | | Papillary (ln) (210) | 46.9 | 1.83 | 7.9 | 0.03 | >300 | >300 |
| 2 | M 67 | Papillary (p) (200) | 56.1 | 3.90 | 4.5 | Uncertain | <10 | 44 |
| 3 | F 19 | Papillary (ln) (210) | 38.7 | 3.39 | 4.4 | Undetectable | <10 | <10 |
| 4 | F 39 | Papillary (ln) (210) | 25.4 | 0.89 | 5.4 | | 20 | 17 |
| 5 | M 60 | Follicular (p) (201) | 19.2 | 0.13 | 25.9 | | <10 | 22 |
| 6 | M 49 | Follicular (ln) (211) | 41.7 | 1.85 | 5.7 | | >300 | >300 |
| Normal thyroid (10) | 20–67 | | 33.6 ±9.9 | 0.99 ±0.50 | 11.2 ±5.4 | | | |

[a] $^{131}$I concentration in tumour, measured at 5 days after administration of the activity, was that observed when T4 had been discontinued and plasma TSH concentration exceeded 30 mU l$^{-1}$. The source of tumour tissue is given in parentheses: p = primary; ln = lymph node. The TNM classification (UICC) for each patient is also included.

**Figure 1** Serum Tg and $^{131}$I concentration during the course of treatment and follow up of patient 1 (**Table I**). The initial operation of total thyroidectomy and removal of lymph nodes (confined to the right side of the neck) preceded $^{131}$I treatment. Radioiodine was concentrated in the lymph node remnant on the right which eventually shrunk sufficiently allowing complete removal (interrupted vertical line).

observed with membranes derived from various other pathological states of the thyroid (Kermode *et al.*, 1984).

The binding affinity and capacity were assessed by measurement of the suppression of binding of $^{125}$I-labelled bTSH in the presence of increasing concentration of non-radioactive bTSH. The binding data were consistent with the 'one binding site' model for TSH-receptor interaction giving a linear Scatchard plot (Scatchard, 1949). There was considerable variation between specimens (Table I) but only with the tissue from one individual (patient 5) was the binding capacity low compared with normal thyroid tissue. The results are presented related to the original tissue weight. Membrane protein content was also determined for these specimens by the method of Lowry *et al.* (1951). The pattern of variability in the binding data was essentially unchanged when expressed relative to membrane protein content.

### *Concentration of $^{131}$I by tumour*

None of the tumours or metastases of the six patients (Table I) had detectable concentrations of $^{131}$I initially when the normal thyroid was present and when plasma TSH levels were within the normal range ($<4\,mU\,l^{-1}$). Following thyroidectomy and withdrawal of any T4 supplement, supranormal levels of plasma TSH were obtained being $>30\,mU\,l^{-1}$ in all patients. Only in patient 1, however, did detectable concentration of $^{131}$I in tumour tissue then occur, localized in metastatic lymph nodes in the neck. None of the other patients developed detectable $^{131}$I uptake in remaining tumour (Table I). The function of tumour tissue of patient 2 could not be determined at supranormal TSH stimulation as operative removal was complete.

### *Thyroglobulin secretion*

These measurements, recorded in Table I, were made in the period just before $^{131}$I concentrating function was determined. All measurements were made after surgical removal of the thyroid and primary tumour, and after an initial $^{131}$I therapy dose to ablate any normal thyroid remnant. In two patients (1 and 6 of Table I), serum Tg was very high and unaffected by T4. Surgical removal of the lymph node remnant after repeated $^{131}$I therapy, abolished both $^{131}$I concentration and Tg secretion (Figure 1). The other patients had undetectable serum Tg when taking T4 but three (patients 2, 4 and 5) had some increase when T4 was withdrawn.

During the period of the present study, 156 patients were attending the follow up clinic, 33 of whom had recurrent tumours in the thyroid bed or in metastases particularly to lymph nodes, bone and lung. Seven of these were follicular and 26 papillary tumours. The serum Tg measurement in these patients was examined in relation to the $^{131}$I concentrating function of the tumours (Figure 2). Concentration of $^{131}$I was undetectable in 8 (23%) of the tumours but the tumours of two patients secreted considerable amounts of Tg. In the remaining patients, although both $^{131}$I concentration and Tg secretion were present, the extent of each function varied considerably from one individual to another (Figure 2); several patients with high serum Tg concentration had tumours which concentrated relatively little $^{131}$I. There were no evident features, clinical or histological, which could be correlated with this variability.

### **Discussion**

A number of studies of TSH receptors have shown that they can be identified in differentiated thyroid tumours although uniformly nonfunctioning tumours such as medullary and anaplastic thyroid carcinomata have none or very few TSH receptors (Takahashi *et al.*, 1978; Carayon *et al.*, 1980; Abe *et*

**Figure 2** The relationship in 33 patients of $^{131}$I concentration by tumour, measured at 5 days after administration of the activity, to the serum Tg concentration. Five patients had neither detectable $^{131}$I concentration nor serum Tg.

*al.*, 1981). On the other hand, papillary and follicular carcinomata vary considerably in their $^{131}$I concentrating ability. Radioiodine concentrating function *in vivo* and TSH receptor complement have not been correlated and it was the particular object of the present study to make this comparison. All the differentiated tumours of our patients possessed clearly demonstrable TSH receptors. The tumour of patient 5 showed the poorest differentiation and was also nonfunctioning. It also had the lowest complement of receptors. Of the other tumours, although only that of patient 1 had measurable $^{131}$I concentrating function, all had similar and apparently normal receptor complements. Patient 1 has been satisfactorily treated with several doses of $^{131}$I but in none of the other patients did this prove possible. In practice, therefore, measurement of TSH receptor complement as a guide to potential tumour function, and thereby to possible future $^{131}$I therapy, is unlikely to be of value. The method does not appear to identify potentially functioning tumours any better than the guide provided by histological appearance.

The other aspect of thyroid cellular function, namely Tg production, was investigated by the serum Tg measurements. The best indicator of tumour production of Tg is probably obtained when serum Tg is measured at the time the patient is taking full doses of T4 since Tg secretion from any normal thyroid remnant should then be completely suppressed (Fui *et al.*, 1979). On this basis, the patients 1, 4 and 6 had evidence of tumour Tg production. In patient 1 her high level of serum Tg was associated with a tumour which concentrated $^{131}$I relatively well although, as indicated by % of $^{131}$I dose concentrated g$^{-1}$ tissue, the concentration was low compared with that of normal thyroid tissue despite enhanced TSH stimulation. In this patient, both aspects of thyroid cell function were evidently present. By contrast in the other two patients, 4 and 6, no $^{131}$I concentrating function was detectable in the tumours although the TSH receptors were well within the normal range in the tumours of these patients. Studies of the iodine content of Tg derived from tumour tissue have shown that it may be very low (Schneider *et al.*, 1983) an observation which, together with the present results, indicates that the retention of Tg secreting activity by the tumour does not necessarily mean that the tumour will concentrate iodine well.

In conclusion, our observations show that the presence of a normal complement of TSH receptors does not indicate that iodine concentration will occur even when plasma TSH levels are much elevated. The mediating factors between the TSH-receptor complex and iodide trapping and organification are presumably inadequate in some tumour cells. Secretion of Tg by tumour tissue, in contrast to normal thyroid tissue, appears to be largely independent of TSH control. Moreover considerable Tg secretion may occur from some tumours which have little or no functional iodine concentrating mechanism even though stimulated by high plasma TSH concentrations. Nevertheless, in the majority of patients, the capacity of the tumour to secrete Tg does appear to indicate that it will also concentrate iodine although the relationship between these two cellular functions varies considerably from one tumour to another.

We gratefully acknowledge Dr P. Byfield and Miss Jennifer Mackenzie for development and application of the thyroglobulin assay, Dr J.G. Pierce for the gift of highly purified bTSH, the National Institute of Biological Standards and Controls, London for hTSH, Professor F.V. Flynn for laboratory facilities and the Cancer Research Campaign for financial support at University College Hospital.

### References

ABE, Y., ICHIKAWA, Y., MURAKI, T. & HOMMA, M. (1981). Thyrotropin (TSH) receptor and adenylate cyclase activity in human thyroid tumours: Absence of high affinity receptor and loss of TSH responsiveness in undifferentiated thyroid carcinoma. *J. Clin. Endocrin. Metab.*, **52,** 23.

CARAYON, P., THOMAS-MORVAN, C., CASTANAS, E. & TUBIANA, M. (1980). Human thyroid cancer: Membrane thrytropin binding and adenylate cyclase activity. *J. Clin. Endocrin. Metab..*, **51,** 915.

CORBETT, B.D., CUNNINGHAM, R.M., HALNAN, K.E. & POCHIN, E.E. (1956). A profile counter and its calibration. *Physics, Med. & Biol.*, **1,** 37.

EDMONDS, C.J., HAYES, S., KERMODE, J.C. & THOMPSON, B.D. (1977). Measurement of serum TSH and thyroid hormones in the management of treatment of thyroid carcinomas with radioiodine. *Br. J. Radiol.*, **50,** 799.

FUI, S.C.N.T., HOFFENBURG, R., MAISEY, M.N. & BLACK, E. (1979). Serum thryoglobulin concentrations and whole-body radioiodine scan in follow-up of differentiated thyroid cancer after thyroid ablation. *Br. Med. J.*, **ii,** 298.

KERMODE, J.C. & THOMPSON, B.D. (1980). Iodination of thyroid-stimulating hormone for receptor-binding studies with human thyroid membranes: Effects of specific activity and method of iodination. *J. Endocrin.*, **84,** 439.

KERMODE, J.C., THOMPSON, B.D. & EDMONDS, C.J. (1981). Comparison of binding of bovine and human thyroid-stimulating hormone to receptor sites on human thyroid membranes. *J. Endocrin.*, **88,** 205.

KERMODE, J.D., EDMONDS, C.J. & MORGANS, M.E. (1984). Receptors for thyroid-stimulating hormones in normal and pathological human thyroid tissues. *J. Endocrin.*, **102,** 369.

LOWRY, O.H., ROSEBROUGH, N.J., FARR, A.L. & RANDALL, R.J. (1951). Protein measurement with Folin phenol reagent. *J. Biol. Chem.*, **193,** 265.

SCATCHARD, G. (1949). The attractions of proteins for small molecules and ions. *Ann. NY. Acad. Sci.*, **51,** 660.

SCHNEIDER, A.B., IKEKUBO, K. & KUMA, K. (1983). Iodine content of serum thyroglobulin in normal individuals and patients with thyroid tumors. *J. Clin. Endocrin. Metab.*, **57,** 1251.

SMITH, N.R. & HALL, R. (1974). Binding of thyroid stimulators to thyroid membranes. *FEBS Lett.*, **42,** 301.

TAKAHASHI, H., JIANG, N.-S., GORMAN, C.A. & LEE, C.Y. (1978). Thyrotropin receptors in normal and pathological thyroid tissues. *J. Clin. Endocrin. Metab.*, **47,** 870.


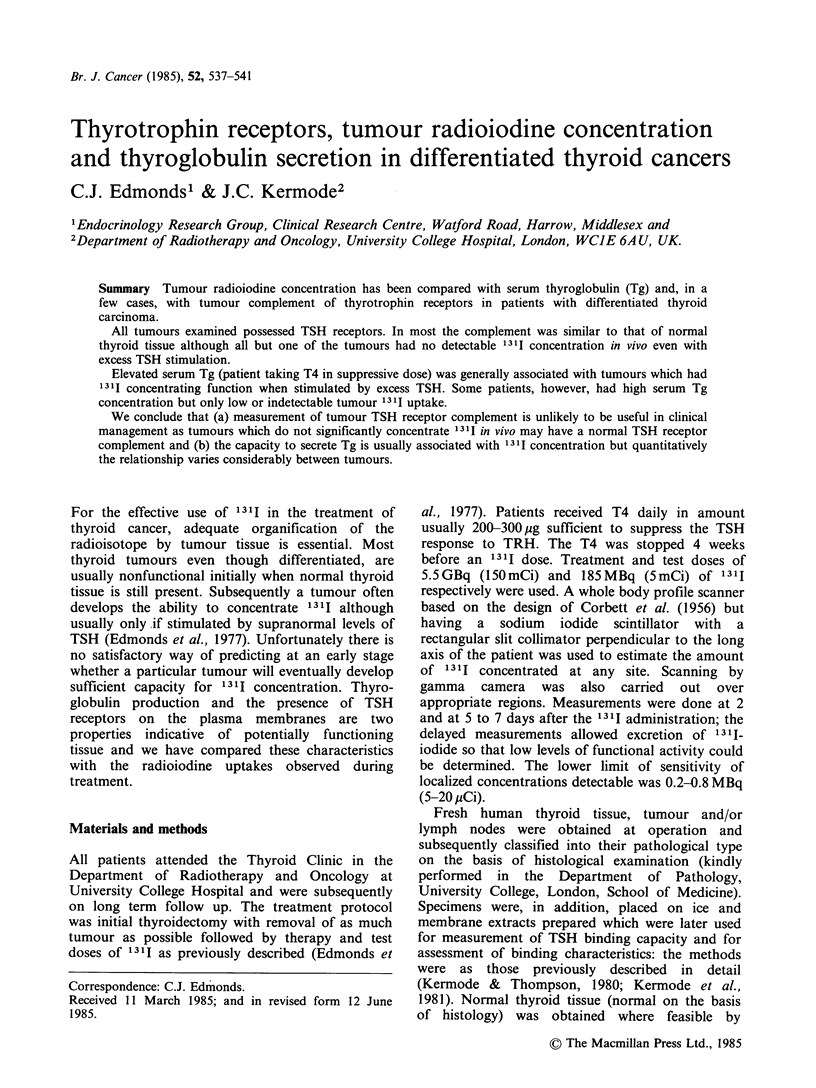

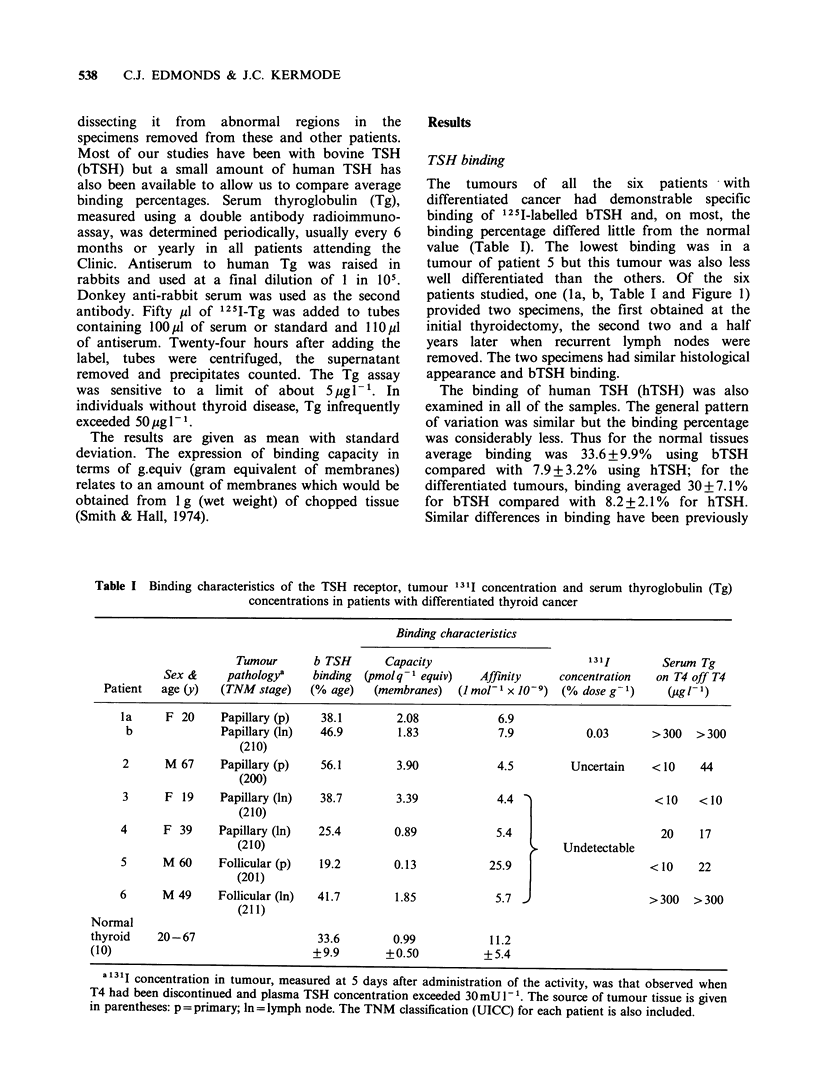

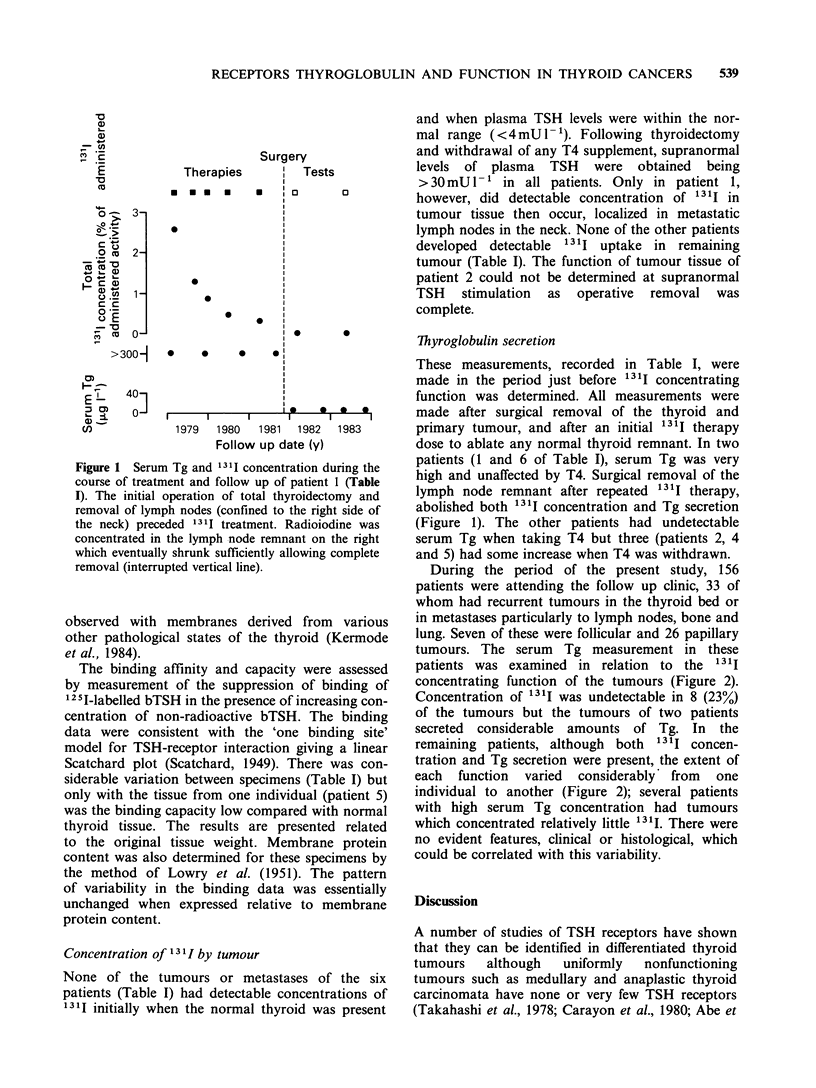

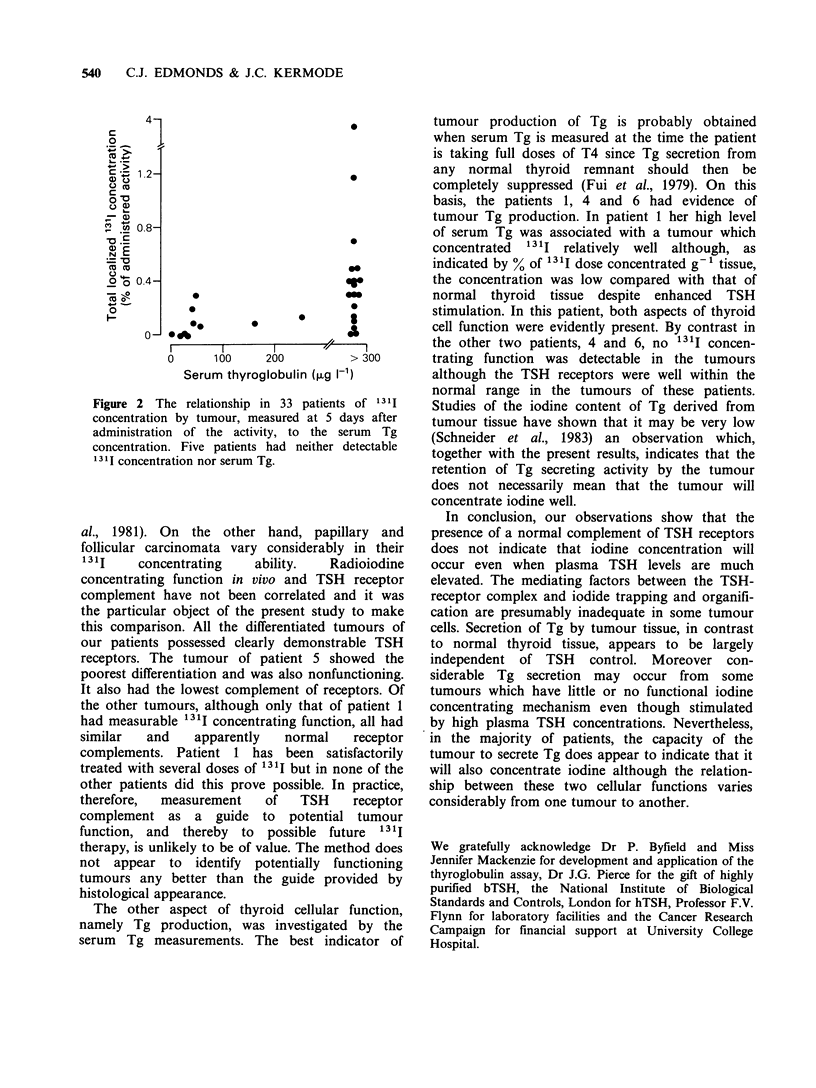

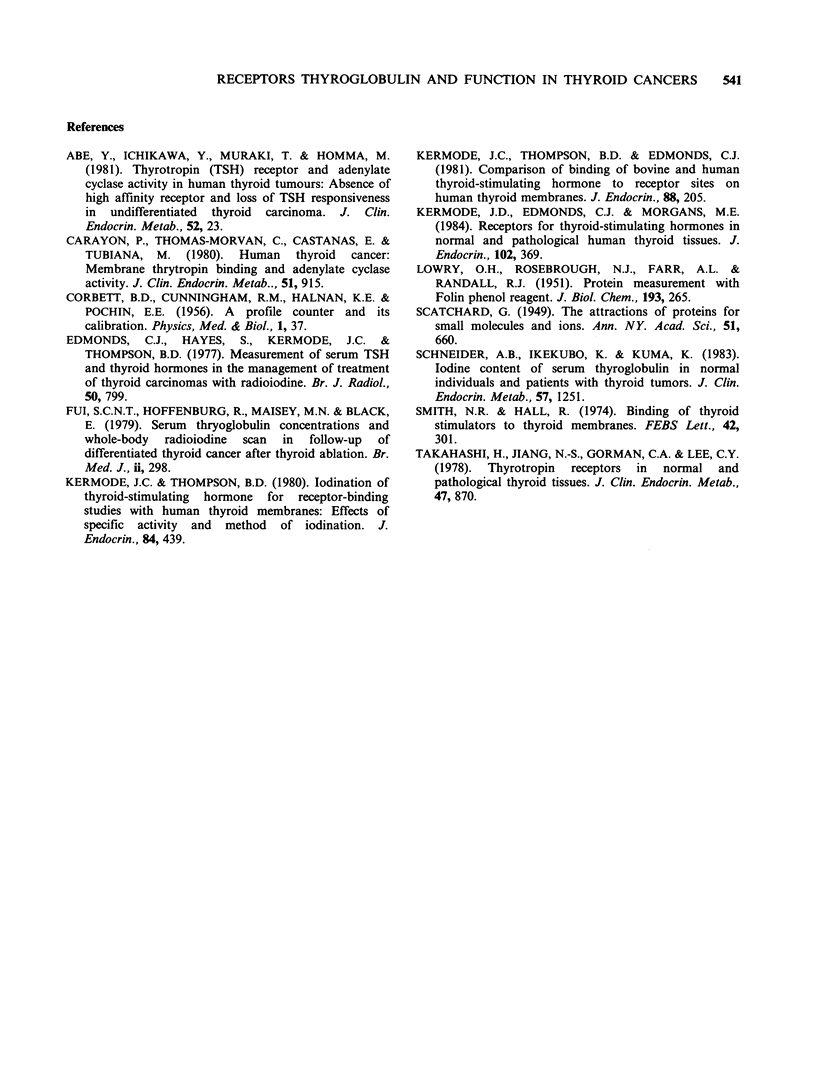


## References

[OCR_00527] Abe Y., Ichikawa Y., Muraki T., Ito K., Homma M. (1981). Thyrotropin (TSH) receptor and adenylate cyclase activity in human thyroid tumors: absence of high affinity receptor and loss of TSH responsiveness in undifferentiated thyroid carcinoma.. J Clin Endocrinol Metab.

[OCR_00535] Carayon P., Thomas-Morvan C., Castanas E., Tubiana M. (1980). Human thyroid cancer: membrane thyrotropin binding and adenylate cyclase activity.. J Clin Endocrinol Metab.

[OCR_00546] Edmonds C. J., Hayes S., Kermode J. C., Thompson B. D. (1977). Measurement of serum TSH and thyroid hormones in the management of treatment of thyroid carcinoma with radioiodine.. Br J Radiol.

[OCR_00573] Kermode J. C., Edmonds C. J., Morgans M. E. (1984). Receptors for thyroid-stimulating hormone in normal and pathological human thyroid tissues.. J Endocrinol.

[OCR_00567] Kermode J. C., Thompson B. D., Edmonds C. J. (1981). Comparison of binding of bovine and human thyroid-stimulating hormone to receptor sites on human thyroid membranes.. J Endocrinol.

[OCR_00560] Kermode J. C., Thompson B. D. (1980). Iodination of thyroid-stimulating hormone for receptor-binding studies with human thyroid membranes: effects of specific activity and method of iodination.. J Endocrinol.

[OCR_00579] LOWRY O. H., ROSEBROUGH N. J., FARR A. L., RANDALL R. J. (1951). Protein measurement with the Folin phenol reagent.. J Biol Chem.

[OCR_00553] Ng Tang Fui S. C., Hoffenberg R., Maisey M. N., Black E. G. (1979). Serum thyroglobulin concentrations and whole-body radioiodine scan in follow-up of differentiated thyroid cancer after thyroid ablation.. Br Med J.

[OCR_00589] Schneider A. B., Ikekubo K., Kuma K. (1983). Iodine content of serum thyroglobulin in normal individuals and patients with thyroid tumors.. J Clin Endocrinol Metab.

[OCR_00595] Smith B. R., Hall R. (1974). Binding of thyroid stimulators to thyroid membranes.. FEBS Lett.

[OCR_00600] Takahashi H., Jiang N. S., Gorman C. A., Lee C. Y. (1978). Thyrotropin receptors in normal and pathological human thyroid tissues.. J Clin Endocrinol Metab.

